# Gut mycobiome dysbiosis in digestive system cancers: immune remodeling and translational implications

**DOI:** 10.3389/fcimb.2026.1902754

**Published:** 2026-10-07

**Authors:** LingBo Ma, Zhen Wang, Jie Mao

**Affiliations:** 1 Lanzhou University, Lanzhou, China; 2 Department of General Surgery, Lanzhou University Second Hospital, Lanzhou, China

**Keywords:** colorectal cancer, fungal dysbiosis, fungal-bacterial interactions, gastric cancer, gut mycobiome, pancreatic ductal adenocarcinoma, tumor immune microenvironment

## Abstract

Fungal dysbiosis has been reported in colorectal cancer (CRC), gastric cancer (GC), and pancreatic ductal adenocarcinoma (PDAC), but evidence strength differs substantially by tumor type and model system. Human CRC studies provide the broadest ecological evidence, including altered fungal community structure and fungal-bacterial networks. Human GC evidence is mainly cross-sectional and identifies *Candida albicans* as a candidate tissue-associated signal rather than a proven driver. In PDAC, human tissue studies report fungal signals, whereas causal support for *Malassezia*–mannose-binding lectin (MBL)–complement and fungal IL-33/type 2 immunity derives principally from mouse perturbation models. This narrative review synthesizes cancer-specific observations, fungal sensing, epithelial injury, immune remodeling, cross-kingdom interactions, and translational implications. We emphasize that low fungal biomass, contamination, transient dietary or environmental fungi, non-standardized sequencing, spatial uncertainty, and reverse causation limit interpretation. Fungal detection does not establish viability, colonization, or tumor causation. Clinical translation will require standardized controls, absolute fungal-load measurements, spatial confirmation, longitudinal cohorts, functional validation, and external testing of whether fungal features add value beyond established clinical, bacterial, metabolic, imaging, and immune markers.

## 1 Introduction

Digestive system cancers, including colorectal cancer (CRC), gastric cancer (GC), and pancreatic ductal adenocarcinoma (PDAC), represent a major component of the global cancer burden and remain associated with substantial mortality despite improvements in surgery, systemic therapy, targeted therapy, and immunotherapy (Bray et al., 2024). Tumor progression in these malignancies cannot be explained solely by tumor-intrinsic genomic alterations. Chronic inflammation, epithelial barrier dysfunction, stromal remodeling, immune escape, metabolic stress, and treatment-related ecological pressure interact with host-associated microorganisms to shape the tumor ecosystem (Belkaid and Hand, 2014; Garrett, 2015; Sepich-Poore et al., 2021). The microbiome has therefore shifted from being regarded as a background environmental factor to a dynamic contributor to tumor initiation, progression, immune regulation, and therapeutic response.

The fungal community (mycobiota) remains underexplored despite its immunological relevance. Fungal beta-glucans, mannans, and chitin can activate Dectin-1/CARD9, complement, and Th17/IL-17 pathways (Brown and Gordon, 2005; Gross et al., 2006; LeibundGut-Landmann et al., 2007; Taylor et al., 2007; Netea et al., 2008; Conti and Gaffen, 2010; Drummond and Brown, 2011; Gaffen et al., 2011; Goodridge et al., 2011; Cui et al., 2013; Plato et al., 2013; Kashem et al., 2015; Galloway-Peña et al., 2024). *Candida albicans* can form hyphae and biofilms, produce candidalysin, and generate acetaldehyde (Gainza-Cirauqui et al., 2013; Mayer et al., 2013; Nobile and Johnson, 2015; Moyes et al., 2016). These properties support mechanistic plausibility from infection biology and *in vitro* systems but do not demonstrate that fungi independently cause human digestive system cancer.

Human studies have begun to define cancer-associated fungal patterns. CRC cohorts show altered fungal communities and fungal-bacterial networks, while GC tissue studies report *Candida*-associated ecological alteration (Coker et al., 2019; Papon et al., 2021; Zhong et al., 2021). In PDAC, increased fungal burden and *Malassezia*-associated signals have been detected in human tissue, but the causal MBL-complement and IL-33/type 2 pathways were established mainly through antifungal depletion, recolonization, or pathway perturbation in mouse models (Aykut et al., 2019; Alam et al., 2022). Pan-cancer studies reinforce cancer-type specificity while also highlighting contamination and spatial uncertainty (Dohlman et al., 2022; Narunsky-Haziza et al., 2022).

Evidence on the esophageal cancer-associated mycobiome remains limited (Rao et al., 2022). Therefore, this review focuses on CRC, GC, and PDAC because these tumor types currently provide the most coherent evidence base for digestive system cancer mycobiome research. Although digestive system cancers also include esophageal, hepatobiliary, small-intestinal, and other tumors, these entities are discussed as emerging or future directions rather than as parallel core sections, reflecting the uneven maturity of available evidence.

We first summarize the healthy gut mycobiome and its role in host steady state, then review cancer-specific fungal dysbiosis, shared mechanisms of immune and epithelial remodeling, and clinical translation. Throughout, we distinguish association from causality and emphasize that fungal dysbiosis should be interpreted within a broader fungal-bacterial-host immune ecosystem rather than as a single-pathogen explanation for cancer.

In this review, “gut mycobiome” is used as an umbrella term for fungal communities detected in stool, mucosal samples, and digestive system tumor-associated compartments, while tissue-associated fungal signals are interpreted separately when spatial localization is relevant. The overall conceptual framework is summarized in **Figure 1**.

**Figure 1:**
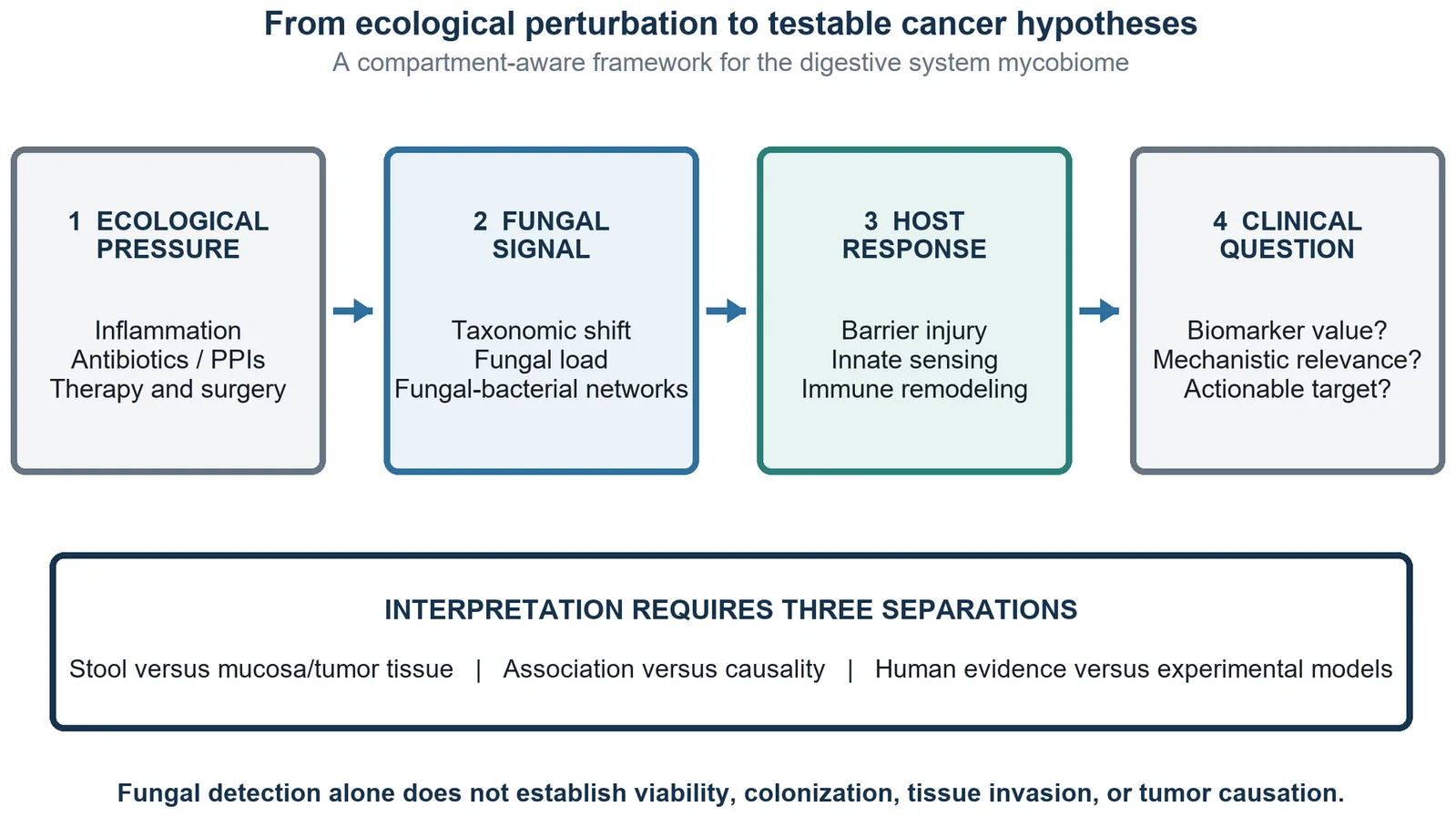
Compartment-aware framework linking ecological perturbation, fungal signals, host responses, and testable clinical questions. Ecological pressures can alter fungal load, composition, and fungal-bacterial networks. These signals may accompany barrier injury and immune remodeling, but detection must be interpreted by anatomical compartment, evidence source, and causal tier. The figure therefore frames biomarker and therapeutic applications as questions requiring validation rather than established clinical uses.

## 2 Literature search and review approach

Literature was searched in PubMed, Web of Science, and Google Scholar using combinations of terms including “mycobiome”, “fungal dysbiosis”, “*Candida*”, “*Malassezia*”, “colorectal cancer”, “gastric cancer”, “pancreatic ductal adenocarcinoma”, “tumor immune microenvironment”, and “microbiome-based therapy”. Priority was given to peer-reviewed original studies, mechanistic reports, pan-cancer analysis, and recent reviews. This article is a narrative review rather than a systematic review.

## 3 The healthy gut mycobiome and host homeostasis

The human gut is dominated by bacteria; fungi are commonly estimated to represent only about 0.1% of total gut microbes, although the estimate depends on extraction efficiency, genome size, sample type, host DNA burden, and analytical pipeline (Zhang et al., 2022). Ascomycota and Basidiomycota are the most frequently reported phyla, with *Candida* and *Saccharomyces* commonly detected in the gastrointestinal tract and *Malassezia*, *Aspergillus*, and *Cladosporium* reported less consistently (Ghannoum et al., 2010; Hoffmann et al., 2013; Huseyin et al., 2017; Nash et al., 2017; Richard and Sokol, 2019; Zhang et al., 2022). Distribution varies along the oral-gastric-intestinal axis and between stool, mucosa, and tumor tissue. Diet, geography, medications, and environmental or skin-associated exposure can contribute transient signals (Ghannoum et al., 2010; Findley et al., 2013); therefore, detection of fungal DNA does not establish viability, stable colonization, metabolic activity, or tissue invasion. Low-biomass tissue claims require extraction blanks, fungal-load quantification, orthogonal detection, and spatial confirmation (Salter et al., 2014; Knight et al., 2018; Eisenhofer et al., 2019; Fletcher et al., 2023).

Anatomical compartment is therefore central to interpretation. Oral and upper gastrointestinal samples commonly contain *Candida* and *Saccharomyces* signals, whereas stool integrates luminal organisms, dietary exposure, and transit through the entire gastrointestinal tract (Ghannoum et al., 2010; Hoffmann et al., 2013; Huseyin et al., 2017; Nash et al., 2017; Richard and Sokol, 2019). Mucosal or tumor-associated profiles may differ from stool because of local oxygen tension, pH, mucus, inflammation, and treatment effects. *Malassezia*, *Aspergillus*, and *Cladosporium* detections are especially vulnerable to skin, environmental, or dietary explanations unless supported by fungal-load measurements, matched controls, and spatial localization. Current data do not define a universal stable fungal core across all healthy individuals or gastrointestinal sites (Ghannoum et al., 2010; Hoffmann et al., 2013; Huseyin et al., 2017; Nash et al., 2017; Richard and Sokol, 2019; Zhang et al., 2022).

Healthy-cohort studies show that gut fungal communities are highly individualized and less stable than bacterial communities (Nash et al., 2017). Diet and co-resident bacteria can shape stool fungal profiles, and these effects may be amplified in cancer by altered nutrition, antibiotics, acid suppression, surgery, chemotherapy, and immunosuppression (Hoffmann et al., 2013; David et al., 2014; Makki et al., 2018).

The host immune system does not ignore intestinal fungi. Fungal cell walls contain beta-glucans, mannans, and chitin that are detected by C-type lectin receptors, Toll-like receptors, complement pathways, and downstream adaptor proteins such as CARD9 (Brown and Gordon, 2005; Gross et al., 2006; Taylor et al., 2007; Netea et al., 2008; Drummond and Brown, 2011; Plato et al., 2013). CX3CR1-positive mononuclear phagocytes can control immunity to intestinal fungi, linking fungal recognition to both innate and adaptive mucosal responses (Leonardi et al., 2018). In physiological settings, these interactions support immune vigilance, barrier maintenance, and containment of opportunistic fungi rather than overt pathology. Th17/IL-17 responses are central to mucosal antifungal defense, especially against *Candida albicans*, but persistent activation of similar circuits may acquire pro-inflammatory or tumor-supportive consequences in chronically inflamed tissues (LeibundGut-Landmann et al., 2007; Conti and Gaffen, 2010; Gaffen et al., 2011; Goodridge et al., 2011; Kashem et al., 2015; Wheeler et al., 2016).

Inflammatory bowel disease provides supporting, not cancer-specific, evidence that barrier dysfunction, fungal dysbiosis, and altered fungal-bacterial relationships can coexist and influence mucosal or systemic immunity (Wheeler et al., 2016; Sokol et al., 2017). Extrapolation to CRC, GC, or PDAC should therefore be treated as biological context rather than direct tumor causality.

Fungal-bacterial interactions include nutrient competition, pH effects, antimicrobial metabolites, physical biofilm structure, and convergent immune signaling (Peleg et al., 2010; Nobile and Johnson, 2015; Jiang et al., 2020). Antibiotic-mediated loss of bacterial colonization resistance can permit *Candida* expansion, but taxonomic co-occurrence alone cannot identify direction or mechanism. Multi-kingdom profiling and spatial or functional experiments are needed.

Methodological interpretation is critical for any cancer mycobiome study. Internal transcribed spacer (ITS) sequencing remains the most common method for fungal profiling, and the ITS region is widely recognized as a fungal DNA barcode (Schoch et al., 2012). However, ITS1/ITS2 primer choice, sequencing depth, reference database coverage, and taxonomic pipelines strongly influence fungal profiles (Nilsson et al., 2019a; Nilsson et al., 2019b). Shotgun metagenomics can profile multiple microbial kingdoms simultaneously, but low fungal abundance, high host DNA content, and incomplete fungal genomes remain important limitations (Knight et al., 2018; Nilsson et al., 2019a; Nilsson et al., 2019b). Culture, quantitative PCR, fluorescence *in situ* hybridization, immunohistochemistry, spatial microbiomics, and strict negative controls are therefore essential for determining whether fungal DNA represents true tissue localization, luminal carryover, or background signal (Salter et al., 2014; Knight et al., 2018; Eisenhofer et al., 2019; Fletcher et al., 2023).

## 4 Mycobiome dysbiosis across digestive system cancers

### 4.1 Colorectal cancer

CRC currently provides the richest clinical evidence base for digestive system cancer-associated fungal dysbiosis. Coker and colleagues demonstrated that CRC is associated with altered fungal community composition, increased Ascomycota/Basidiomycota ratio, altered fungal ecological networks, and candidate fungal markers that may help distinguish CRC from healthy controls (Coker et al., 2019). *Candida*, *Saccharomyces*, *Trichosporon*, *Malassezia*, and *Aspergillus*-related signatures have been reported in different studies, but *Candida* remains the most consistently discussed genus. These observations should be interpreted carefully because stool and mucosal fungal profiles are affected by geography, diet, medication exposure, sequencing region, and background contamination (Schoch et al., 2012; Salter et al., 2014; Huseyin et al., 2017; Nash et al., 2017; Eisenhofer et al., 2019; Nilsson et al., 2019a; Nilsson et al., 2019b; Richard and Sokol, 2019). Nevertheless, the reproducible observation that fungal ecology differs between CRC patients and controls supports the idea that fungal dysbiosis is a meaningful feature of the CRC microbial ecosystem.

The biological interpretation of CRC-associated fungi is still evolving. *Candida albicans* and *Candida tropicalis* can adhere to epithelial surfaces, form biofilms, transition to invasive hyphal forms, and activate mucosal immune pathways (Mayer et al., 2013; Nobile and Johnson, 2015). These features provide plausible links between fungal expansion, epithelial injury, and chronic inflammation. However, most human CRC mycobiome studies remain observational, and it would be premature to classify *Candida* or other enriched fungi as established CRC drivers. A more appropriate interpretation is that fungi may act as ecological amplifiers, inflammatory modulators, or biomarkers within a broader tumor-associated microenvironment. This distinction is important because the CRC microbiome already includes multiple bacterial mechanisms with stronger causal evidence, including *Fusobacterium nucleatum*-mediated signaling, biofilm organization, genotoxic *Escherichia coli*, and inflammatory microbial products (Arthur et al., 2012; Grivennikov et al., 2012; Kostic et al., 2013; Rubinstein et al., 2013; Dejea et al., 2014; Gur et al., 2015; Abed et al., 2016; Mima et al., 2016; Bullman et al., 2017; Yu T. et al., 2017; Dejea et al., 2018; Kadosh et al., 2020; Pleguezuelos-Manzano et al., 2020).

Cross-kingdom ecological remodeling is a particularly important feature of CRC. Coker and colleagues reported disruption of fungal-bacterial networks in CRC (Coker et al., 2019), while Dejea and colleagues showed that polymicrobial biofilms are a spatial feature of proximal colorectal cancers and can be associated with epithelial barrier disruption and increased proliferation (Dejea et al., 2014). In familial adenomatous polyposis, colonic biofilms containing tumorigenic bacteria have been described, illustrating how microbial spatial organization may precede or accompany neoplastic progression (Dejea et al., 2018). Although these studies do not prove that fungi are essential components of CRC biofilms, *Candida* biology makes fungal participation plausible. Therefore, CRC fungal dysbiosis should be integrated with bacterial ecology, epithelial barrier status, inflammatory signaling, and spatial organization rather than treated as an isolated taxonomic shift.

The immune relevance of CRC fungal dysbiosis is supported primarily by preclinical evidence. In a mouse colon tumor model, Wang and colleagues showed that CARD9 protects against tumorigenesis by restricting mycobiota-mediated expansion of myeloid-derived suppressor cells (MDSCs) (Wang et al., 2018). This experiment links fungal dysbiosis, innate immune adaptor signaling, and immunosuppressive myeloid remodeling, but it should not be interpreted as proof of the same causal pathway in patients. Human CRC studies remain predominantly observational; paired longitudinal profiling of fungal taxa, CARD9/Dectin-1 signaling, myeloid states, epithelial barrier defects, and bacterial networks is needed for clinical validation.

### 4.2 Gastric cancer

Gastric cancer represents a distinct upper gastrointestinal ecological niche in which fungal dysbiosis must be interpreted against the dominant role of *Helicobacter pylori*, gastric acid, mucosal atrophy, intestinal metaplasia, bile reflux, medication exposure, and regional anatomy. *H. pylori* remains a core carcinogenic risk factor and can drive chronic gastritis, epithelial injury, atrophy, metaplasia, and neoplastic transformation (Polk and Peek, 2010; Amieva and Peek, 2016). Gastric bacterial dysbiosis also accompanies gastric carcinogenesis, indicating that tumor-associated ecological change extends beyond *H. pylori* alone (Coker et al., 2018). Within this context, fungal studies are emerging but remain less mature than CRC bacterial or PDAC fungal studies.

Zhong and colleagues reported that gastric cancer tissues exhibit altered fungal community structure and identified *Candida albicans* as a candidate fungus associated with gastric carcinogenesis (Zhong et al., 2021). Papon and colleagues emphasized that this finding provides an important clue but does not establish causality (Papon et al., 2021). At the genus level, *Candida* and *Alternaria* were reported as relatively enriched in tumor tissue, while other taxa showed differential patterns; at the species level, *C. albicans* was the most prominent candidate marker. These observations support the presence of gastric fungal ecological remodeling, but the evidence remains mainly cross-sectional and tissue-based. Larger multicenter studies, paired non-malignant mucosal controls, gastric fluid or brush samples, and spatial validation are needed before fungal signatures can be considered clinically actionable in GC.

The biological plausibility of *C. albicans* in the gastric niche derives from its ability to adhere, form biofilms, generate hyphae, injure epithelium, and activate immune pathways (Mayer et al., 2013; Nobile and Johnson, 2015). However, gastric cancer is not a candidiasis model. *C. albicans* enrichment may reflect a permissive tumor niche created by reduced acidity, mucosal injury, altered nutrients, or inflammation rather than a primary carcinogenic event. This nuance is essential for a high-quality review: the best-supported statement is not that *C. albicans* causes gastric cancer, but that *C. albicans* is a candidate fungal marker and potential ecological participant in gastric mucosal remodeling. Its direct functional role requires longitudinal sampling, tissue localization, culture or quantitative validation, and experimental modeling.

*H. pylori*-*Candida* interactions provide an additional but still exploratory framework. *In vitro* studies show acidic-stress incorporation, co-aggregation, and dual-species biofilm formation, while a mouse gastritis model suggests that fungal vacuoles may protect *H. pylori* under adverse conditions (Sánchez-Alonzo et al., 2020; Chen et al., 2021; Hiengrach et al., 2022; Palencia et al., 2022). These experimental observations support biological plausibility but do not demonstrate that *H. pylori*-*C. albicans* interactions cause human gastric cancer. Human studies should therefore stratify *H. pylori* status, virulence factors, gastric region, acid suppression, intestinal metaplasia stage, and immune context.

### 4.3 Pancreatic ductal adenocarcinoma

PDAC provides the strongest experimental evidence for a tumor-promoting fungal pathway among digestive system cancers. In mouse PDAC models, Aykut and colleagues used antifungal depletion, recolonization, and immune-pathway perturbation to show that gut-derived fungi can influence tumor progression through mannose-binding lectin (MBL) activation (Aykut et al., 2019). The same study detected increased fungal burden and *Malassezia* enrichment in human PDAC tissue, but those human observations establish association and presence rather than independent causality. This separation between human tissue evidence and mouse causal evidence is essential when interpreting the gut-pancreas axis.

The *Malassezia*–mannose-binding lectin (MBL)–complement axis is currently the most clearly defined fungal mechanism in experimental PDAC. In mouse models, *Malassezia*-associated activation of complement component C3 and signaling through C3a and the C3a receptor (C3aR) contributed to tumor progression, whereas disruption of MBL, C3, or C3aR attenuated the effect (Aykut et al., 2019). These perturbation data support causality within the model; confirmation of pathway activity, spatial localization, and prognostic relevance in independent human cohorts remains necessary. Complement can support antimicrobial defense or, depending on context, inflammation, vascular remodeling, myeloid recruitment, and immune suppression (Afshar-Kharghan, 2017; Reis et al., 2018).

A second PDAC mechanism, established in experimental models, involves IL-33 and type 2 immunity. Alam and colleagues showed that tumor-associated fungal communities can induce IL-33 secretion and type 2 immune responses in pancreatic cancer models (Alam et al., 2022). IL-33 can recruit Th2 cells and group 2 innate lymphoid cells and promote IL-4-, IL-5-, and IL-13-associated programs (Liew et al., 2016; Gieseck et al., 2018). In the fibrotic PDAC microenvironment, this pathway may reinforce stromal remodeling and immune suppression (Gieseck et al., 2018; Ho et al., 2020; Alam et al., 2022), but its prevalence and clinical importance in patients remain unresolved.

The PDAC field also illustrates why evidence must remain methodologically cautious. Pancreatic tissue is a low-biomass site, so apparent intratumoral fungi may reflect true localization, luminal carryover, reagent contamination, or analytical artifacts. Fletcher and colleagues emphasized this uncertainty in a methodological reassessment (Fletcher et al., 2023). Accordingly, human tissue claims require extraction blanks, batch controls, absolute fungal-load measurements, orthogonal detection, and *in situ* localization. The defensible conclusion is that fungi can influence pancreatic tumor biology in experimental models, whereas their prevalence, spatial distribution, and clinical relevance in patients remain unsettled.

Across tumor types, the evidence gradient is heterogeneous: CRC offers the broadest human ecological evidence but limited causal validation; GC provides a *Candida*-associated mucosal signal that remains predominantly cross-sectional; and PDAC offers the deepest experimental perturbation evidence while posing the greatest low-biomass challenge. These differences preclude assigning the same biological meaning to a taxon across anatomical sites and argue for cancer-specific interpretation of detection, association, spatial plausibility, and causality.

Esophageal, biliary, and small-intestinal tumors remain future directions because evidence is insufficient for parallel core sections. **Figure 2** summarizes the cancer-specific anatomical niches, dominant fungal signals, evidence sources, and principal limitations, while **Tables 1**, **2** provide representative studies, taxa, and cautions.

**Figure 2:**
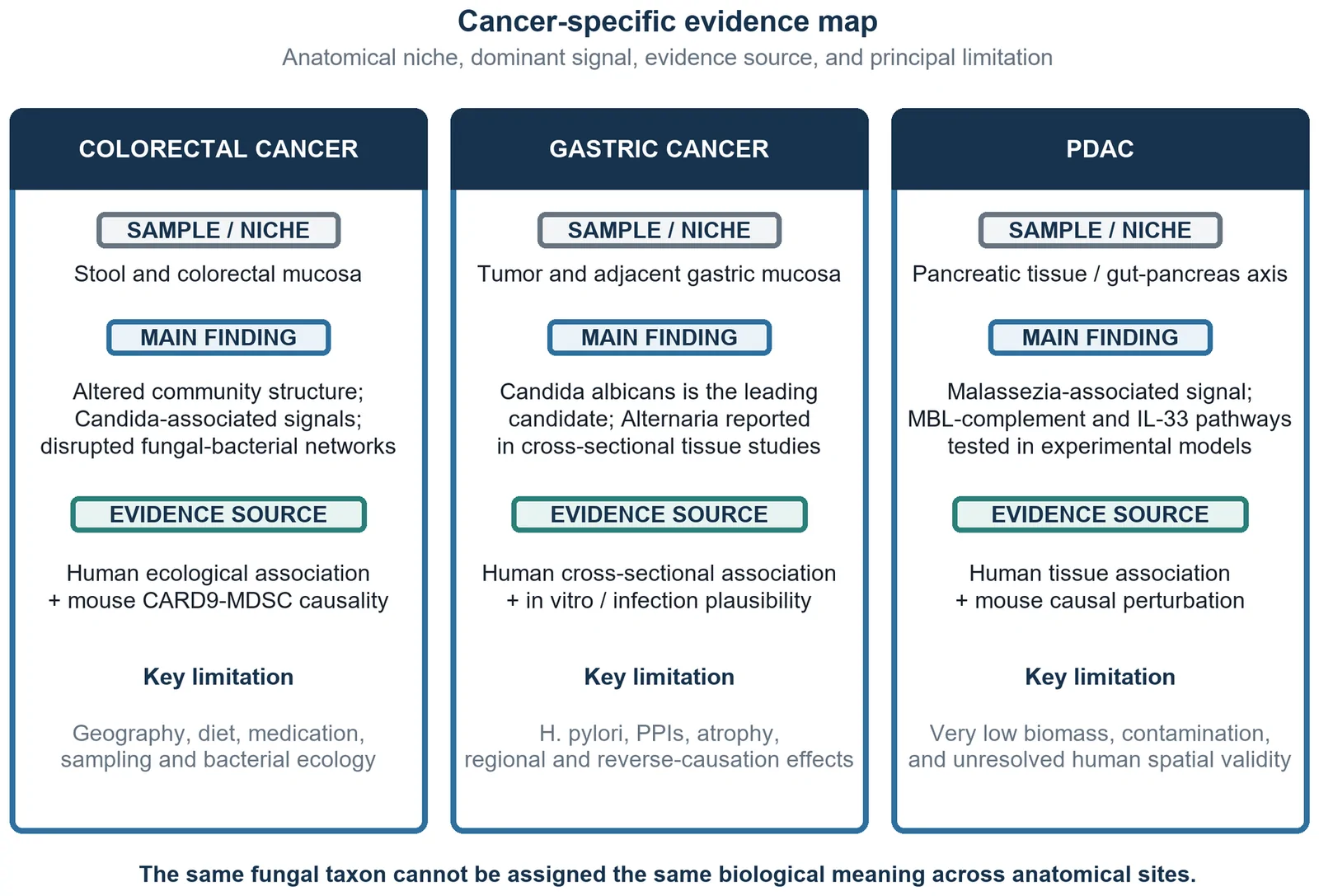
Cancer-specific mycobiome evidence across colorectal cancer, gastric cancer, and pancreatic ductal adenocarcinoma. CRC and GC findings remain largely human observational associations. PDAC combines human tissue association with causal perturbation in mouse models, but human spatial and pathway-level validation remains incomplete. Across all three cancers, anatomical compartment, low biomass, treatment exposure, geography, diet, and bacterial ecology can change the interpretation of the same fungal signal.

**Table 1:** Key studies of mycobiome dysbiosis in digestive system cancers.

Study	Cancer	Sample	Method	Main finding	Interpretation	Evidence source
Coker et al., 2019	CRC	Stool/intestine	ITS; network analysis	Altered fungal structure and fungal-bacterial networks	CRC ecological dysbiosis; candidate adjunct biomarker	Human observational
Wang et al., 2018	Colon tumor model	Mouse intestine	CARD9 model; immune analysis	CARD9 restricted mycobiota-mediated MDSC expansion	Direct fungal-immune mechanism in mice	Mouse causal
Zhong et al., 2021	GC	Tumor/adjacent tissue	ITS tissue profiling	Altered fungal ecology; *C. albicans* leading candidate	Cross-sectional association; causality unproven	Human observational
Aykut et al., 2019	PDAC	Human tissue; mice	Depletion, recolonization, pathway blockade	*Malassezia*–mannose-binding lectin (MBL)–complement promoted tumor growth	Human association plus mouse causality	Mixed human/mouse
Alam et al., 2022	PDAC	Tumor models	Fungal perturbation; immune profiling	Fungi induced IL-33 and type 2 immunity	Tumor-immune mechanism in experimental models	Mouse experimental
Narunsky-Haziza et al., 2022	Pan-cancer	Multiple tumor types	Multi-omics analysis	Cancer-type-specific fungal ecologies and bacterial interactions	Broad association requiring tumor-specific validation	Human observational
Dohlman et al., 2022	Pan-cancer	GI and lung tumors	Pan-cancer profiling	Fungal signals associated with tumor and immune variables	Cross-cancer association; contamination controls essential	Human observational
Fletcher et al., 2023	PDAC methods	Low-biomass tissue	Methodological reassessment	Questioned localization versus background signal	Supports orthogonal and spatial validation	Methodological critique

CRC, colorectal cancer; GC, gastric cancer; PDAC, pancreatic ductal adenocarcinoma; ITS, internal transcribed spacer; MBL, mannose-binding lectin; MDSC, myeloid-derived suppressor cell.

**Table 2:** Fungal taxa and proposed roles across CRC, GC, and PDAC.

Fungal taxon	Tumor type/niche	Reported alteration	Proposed role	Evidence and key caution	Representative references
*Candida albicans*	GC; CRC mucosal niche	Enriched in GC tissue; recurrent *Candida*-associated CRC signal	Adhesion, hyphae, biofilm, candidalysin and epithelial stress	Human association plus infection/*in vitro* plausibility; no proven human cancer causality	(Gainza-Cirauqui et al., 2013; Mayer et al., 2013; Nobile and Johnson, 2015; Moyes et al., 2016; Coker et al., 2019; Papon et al., 2021; Zhong et al., 2021)
*Candida tropicalis*	CRC	Reported in selected CRC profiles	*Candida*te inflammatory or biofilm-associated participant	Human observational association; evidence weaker than for *C. albicans*	(Coker et al., 2019)
*Malassezia*	PDAC; gut-pancreas axis	Detected in PDAC tissue; enriched in experimental models	MBL-C3/C3aR complement activation	Human tissue association plus mouse causal perturbation; low-biomass validation required	(Aykut et al., 2019; Fletcher et al., 2023)
*Trichosporon*	CRC	Differential detection in CRC	*Candida*te ecological marker	Human observational association; no tumor-specific mechanism established	(Coker et al., 2019)
*Aspergillus*	Gut/CRC reports	Variable differential abundance	Possible ecological or dietary signal	Detection may be transient or environmental; colonization not established	(Hoffmann et al., 2013; Huseyin et al., 2017; Nash et al., 2017; Coker et al., 2019; Richard and Sokol, 2019)
*Saccharomyces*	Gut/CRC reports	Variable abundance across cohorts	Commensal, dietary or ecological marker	Strong dietary influence; disease-specific interpretation is uncertain	(Hoffmann et al., 2013; Huseyin et al., 2017; Nash et al., 2017; Coker et al., 2019; Richard and Sokol, 2019)
*Alternaria*	GC tissue	Reported enrichment in GC tissue	*Candida*te tissue-associated marker	Single-cohort human association; requires replication and controls	(Zhong et al., 2021)
Fungal-bacterial network	CRC; pan-cancer	Altered cross-kingdom correlations	Potential biofilm, metabolic and immune co-regulation	Network inference is not causality; spatial and functional validation required	(Peleg et al., 2010; Coker et al., 2019; Sánchez-Alonzo et al., 2020; Chen et al., 2021; Dohlman et al., 2022; Hiengrach et al., 2022; Narunsky-Haziza et al., 2022; Palencia et al., 2022)

CRC, colorectal cancer; GC, gastric cancer; PDAC, pancreatic ductal adenocarcinoma; MBL, mannose-binding lectin; C3aR, complement component 3a receptor.

## 5 Shared mechanisms linking fungal dysbiosis to tumor progression

### 5.1 Fungal sensing and innate immune activation

The first mechanistic layer linking fungal dysbiosis to digestive system tumor biology is immune sensing. Fungal beta-glucans, mannans, and chitin are recognized by pattern-recognition receptors (PRRs) expressed by epithelial cells, macrophages, dendritic cells, neutrophils, and mononuclear phagocytes (Brown and Gordon, 2005; Netea et al., 2008; Underhill and Iliev, 2014; Iliev and Leonardi, 2017). Dectin-1 is a major beta-glucan receptor and signals through Syk, CARD9, NF-κB, and related pathways to induce cytokine production and antifungal immunity (Gross et al., 2006; LeibundGut-Landmann et al., 2007; Taylor et al., 2007; Drummond and Brown, 2011; Goodridge et al., 2011; Plato et al., 2013). In infection, these pathways are protective. In chronic inflammatory or tumor contexts, however, repeated or dysregulated fungal sensing may sustain cytokine release, myeloid recruitment, epithelial proliferation, and immune deviation.

CARD9 is particularly relevant because it bridges fungal recognition and intestinal tumor immunity. Gross and colleagues established CARD9 as a central antifungal signaling adaptor (Gross et al., 2006), whereas Wang and colleagues used a mouse colon tumor model to link CARD9 deficiency to mycobiota-mediated MDSC expansion (Wang et al., 2018). The latter provides experimental causality in mice, not direct evidence that this pathway determines human CRC. Human studies must test whether fungal load and CARD9-associated myeloid states co-localize and predict outcome independently of treatment and bacterial ecology.

Complement adds a second innate sensing layer. In mouse PDAC models, *Malassezia* activated the MBL-mediated lectin pathway and downstream C3a/C3aR signaling, promoting tumor growth (Aykut et al., 2019). These intervention data establish a causal mechanism in the experimental system; corresponding pathway activation has not yet been established as a general mechanism in patients. Complement biology is context-dependent and can support microbial defense or promote inflammation, angiogenesis, myeloid recruitment, and immunosuppression (Afshar-Kharghan, 2017; Reis et al., 2018).

### 5.2 Barrier injury, chronic inflammation, and epithelial remodeling

Fungal dysbiosis may contribute to barrier injury and chronic inflammation, but the relevant evidence is largely mechanistic fungal biology rather than direct human cancer causality. *Candida albicans* can transition to hyphae, adhere, invade, and form biofilms (Mayer et al., 2013; Nobile and Johnson, 2015); candidalysin damages epithelial membranes in mucosal infection models (Moyes et al., 2016), and *Candida* can generate acetaldehyde in oral disease models (Gainza-Cirauqui et al., 2013). These findings support biological plausibility for epithelial stress in susceptible niches but do not show that *Candida* independently initiates CRC or GC in patients.

Barrier defects are important because they convert microbial signals into tissue-level inflammation. In intestinal tumor models, barrier disruption and microbial products can drive IL-23/IL-17-mediated tumor growth (Grivennikov et al., 2012). In CRC, tumor-associated biofilms are linked to epithelial disruption and increased proliferation (Dejea et al., 2014; Dejea et al., 2018). In GC, gastric atrophy, acid suppression, *H. pylori*-related inflammation, and mucosal remodeling may create a niche in which fungi are more likely to persist (Polk and Peek, 2010; Amieva and Peek, 2016; Massarrat et al., 2016; Ferreira et al., 2018; Zhong et al., 2021). In PDAC, ductal obstruction, fibrosis, local hypoxia, and tumor necrosis may create microenvironments that support microbial persistence and immune activation (Pushalkar et al., 2018; Riquelme et al., 2019; Ho et al., 2020; Alam et al., 2022). These site-specific features suggest that fungal dysbiosis should be conceptualized as a niche-dependent process: the same organism may be harmless, transient, or pathogenic depending on epithelial integrity and immune context.

Bacterial studies further demonstrate how microbes can induce epithelial remodeling and carcinogenic processes. Genotoxic pks+ *Escherichia coli* can leave a mutational signature in colorectal cancer (Pleguezuelos-Manzano et al., 2020), and intestinal inflammation can enhance the cancer-inducing activity of the microbiota (Arthur et al., 2012). Although comparable direct fungal mutational signatures have not been established in digestive system tumors, fungal metabolites and fungal-driven inflammation may contribute to oxidative stress, epithelial repair programs, and tissue remodeling. The field should therefore move beyond taxonomic lists toward mechanistic endpoints such as epithelial barrier integrity, DNA damage markers, cell proliferation, organoid responses, and spatial relationships between fungi and epithelial injury.

### 5.3 Immune remodeling and immunosuppressive tumor microenvironments

Fungal dysbiosis can reshape the tumor immune microenvironment through several interconnected pathways. Th17/IL-17 signaling is central to antifungal mucosal defense, especially against *Candida albicans* (Conti and Gaffen, 2010; Gaffen et al., 2011; Kashem et al., 2015). In physiological settings, this pathway promotes epithelial defense and neutrophil recruitment. In chronically inflamed or neoplastic tissues, persistent IL-17 signaling may support inflammation, angiogenesis, epithelial proliferation, and tumor-supportive immune remodeling (Grivennikov et al., 2012). This duality is crucial: antifungal immunity is not inherently anti-tumor immunity, and the outcome of fungal sensing depends on tissue context, receptor engagement, cytokine balance, and the composition of myeloid and lymphoid cells.

Myeloid remodeling is another important route. In mouse colon tumor experiments, CARD9-dependent restriction of mycobiota-mediated MDSC expansion provides a direct fungal-immune link (Wang et al., 2018). Tumor-associated macrophages influence growth, angiogenesis, matrix remodeling, and immune suppression (Martinez and Gordon, 2014; Noy and Pollard, 2014; Veglia et al., 2021), but evidence that specific fungi independently polarize macrophages in human digestive cancers remains limited. Fungal products, complement fragments, IL-33/type 2 immunity, and bacterial co-signals should therefore be evaluated together rather than inferred from taxonomy alone (Afshar-Kharghan, 2017; Gieseck et al., 2018; Ho et al., 2020; Alam et al., 2022).

PDAC illustrates, in experimental models, the potential of fungi to induce non-classical immune programs. Fungal induction of IL-33 and type 2 immunity can recruit Th2 cells and group 2 innate lymphoid cells and may reinforce remodeling and fibrosis (Gieseck et al., 2018; Reis et al., 2018; Alam et al., 2022). Although immune exclusion, regulatory T-cell activity, and T-cell exhaustion are established tumor-context concepts (Wherry and Kurachi, 2015; Kleeff et al., 2016; Binnewies et al., 2018; Neoptolemos et al., 2018; Togashi et al., 2019; Ho et al., 2020), direct human evidence that fungi independently drive these states in digestive system cancers remains limited.

### 5.4 Cross-kingdom microbial networks

Cross-kingdom interaction can occur through nutrient competition, metabolic exchange, adhesion, biofilms, and immune co-regulation (Peleg et al., 2010). Human CRC studies report altered fungal-bacterial networks, but network inference does not establish direct interaction (Coker et al., 2019). *H. pylori*-*C. albicans* co-aggregation and dual-species biofilms are supported mainly by *in vitro* and mouse infection studies (Sánchez-Alonzo et al., 2020; Chen et al., 2021; Hiengrach et al., 2022; Palencia et al., 2022), and PDAC bacterial and fungal signals may converge on immune pathways without proving that they occupy the same human tissue niche (Geller et al., 2017; Pushalkar et al., 2018; Aykut et al., 2019; Riquelme et al., 2019; Alam et al., 2022).

Metabolic crosstalk remains underdeveloped in digestive system cancer. Fungi respond to nutrients, oxygen, pH, lactate, host lipids, and bacterial metabolites, while fungal metabolites can alter epithelial and immune function (Peleg et al., 2010; Louis et al., 2014; Nobile and Johnson, 2015; Mager et al., 2020; Seelbinder et al., 2023). However, relative-abundance data cannot establish metabolite production or directionality. Integrated metagenomic, metatranscriptomic, metabolomic, and spatial measurements are needed to connect fungal activity to host response.

Functional validation of cross-kingdom networks should move beyond correlation. *Candida*te interactions should be tested through defined co-cultures, organoids, gnotobiotic or depletion-reconstitution models, and pathway blockade, with attention to fungal strain, bacterial partner, dose, anatomical site, and treatment context (Peleg et al., 2010; Dejea et al., 2014; Nobile and Johnson, 2015; Coker et al., 2019; Sánchez-Alonzo et al., 2020; Chen et al., 2021; Dohlman et al., 2022; Hiengrach et al., 2022; Narunsky-Haziza et al., 2022; Palencia et al., 2022). A reproducible network may be useful as a biomarker even when its individual edges are not causal, but therapeutic claims require perturbation evidence and confirmation that the organisms or their products occupy the relevant human niche.

A four-tier hierarchy prevents causal overstatement: Tier 1, detection without demonstrated viability; Tier 2, reproducible association after confounder control; Tier 3, spatial and functional plausibility; and Tier 4, causal perturbation through depletion, recolonization, dose response, or pathway blockade. Most human CRC and GC findings remain at Tiers 1–2, with selected evidence approaching Tier 3. PDAC *Malassezia*-MBL and fungal IL-33 pathways reach Tier 4 in mouse models, not yet in patients (Aykut et al., 2019; Zhong et al., 2021; Alam et al., 2022; Dohlman et al., 2022; Narunsky-Haziza et al., 2022; Fletcher et al., 2023). Every mechanistic claim should therefore identify compartment, model system, and evidence tier. These pathways are summarized in **Figure 3**.

**Figure 3:**
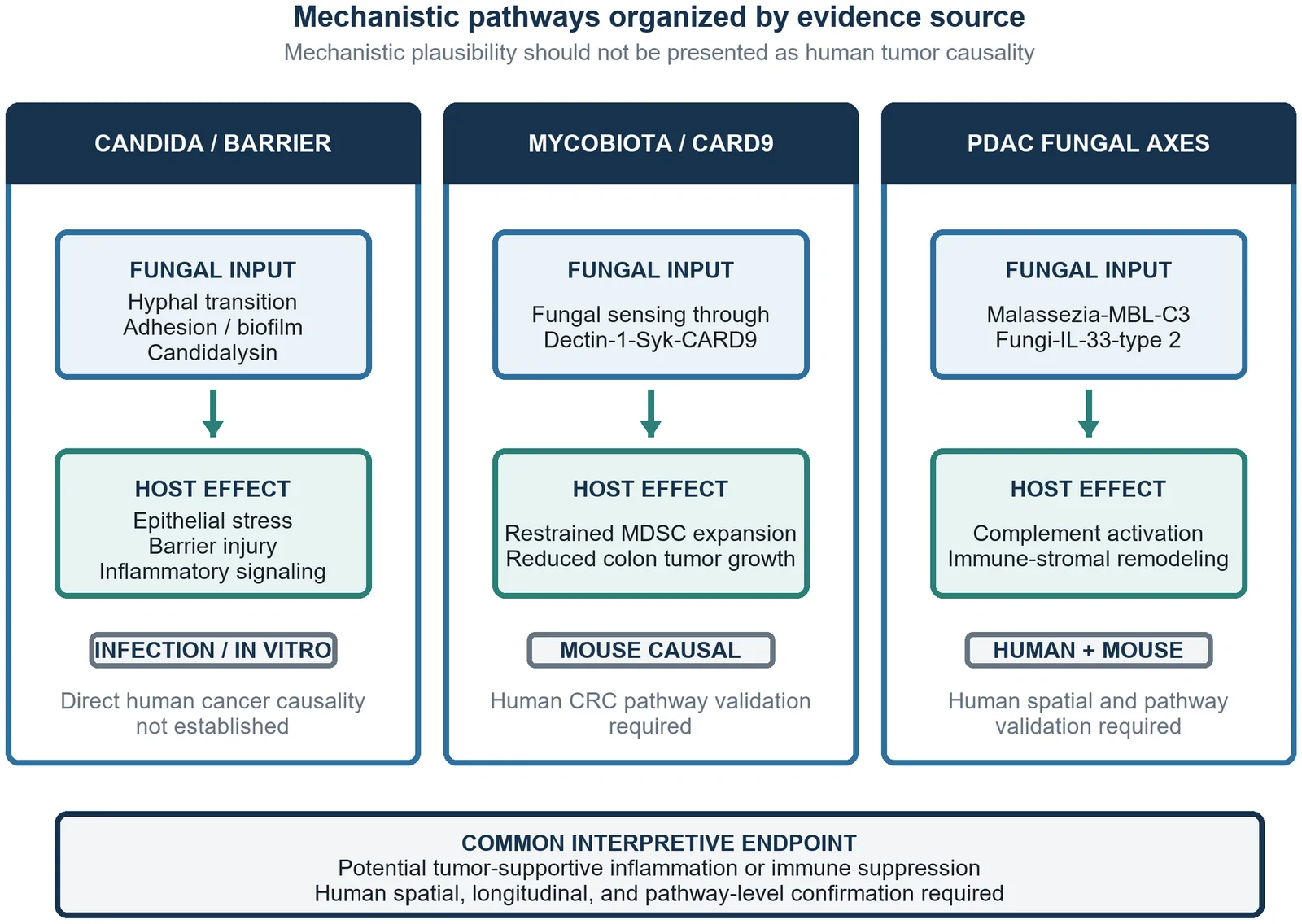
Mechanistic pathways organized by evidence source. *Candida*-associated barrier effects are supported mainly by infection and *in vitro* biology; CARD9-MDSC and *Malassezia*-MBL/IL-33 pathways have causal support in mouse tumor models. Human cancer evidence remains predominantly associative and requires spatial, longitudinal, and pathway-level confirmation.

## 6 Clinical translation

### 6.1 Diagnosis and risk stratification

Fungal signatures may complement, but cannot yet replace, established diagnostic tools. CRC stool is the most accessible setting, but studies developing candidate models should enroll pretreatment cohorts, test geographically independent populations, and report absolute fungal load, key medications, diet, and bacterial ecology. They must demonstrate discrimination, calibration, and clinical net benefit beyond fecal immunochemical testing, colonoscopic risk factors, bacterial signatures, and metabolomics (Zeller et al., 2014; Yu J. et al., 2017; Thomas et al., 2019; Wirbel et al., 2019; Dohlman et al., 2022; Narunsky-Haziza et al., 2022). Because many stool fungi are transient and fungal features vary across cohorts, reproducible multi-kingdom profiles may be more credible than a single-taxon classifier. Current evidence does not support standalone fungal screening.

In GC, *C. albicans* is a candidate tissue-associated marker, but biopsy signals are influenced by *H. pylori*, atrophy, intestinal metaplasia, acid suppression, anatomy, oral carryover, and medication (Polk and Peek, 2010; Amieva and Peek, 2016; Massarrat et al., 2016; Coker et al., 2018; Zhong et al., 2021). Any diagnostic study should separate tumor from adjacent mucosa and benign inflammatory controls and should confirm fungal load and localization. In PDAC, tissue access limits screening, whereas fecal multi-omic signatures may be more practical; fungal features nevertheless require independent testing against CA 19-9, imaging, cyst characteristics, pancreatitis, and clinical risk factors (Aykut et al., 2019; Riquelme et al., 2019; Kartal et al., 2022). Existing GC and PDAC results remain discovery-stage.

The most informative risk-stratification studies would enroll defined high-risk populations before cancer develops, such as patients with advanced colorectal neoplasia, gastric premalignant lesions, pancreatic cysts or chronic pancreatitis, and inherited or familial risk. Repeated sampling could determine whether fungal change precedes neoplasia, improves risk classification, or merely tracks inflammation, obstruction, diet, and treatment. External validation, calibration, decision-curve analysis, and prespecified clinical thresholds are more informative than abundance differences alone (Zeller et al., 2014; Yu J. et al., 2017; Aykut et al., 2019; Thomas et al., 2019; Wirbel et al., 2019; Zhong et al., 2021; Kartal et al., 2022).

### 6.2 Prognosis and disease monitoring

The prognostic evidence is weaker than the diagnostic evidence. Pan-cancer fungal associations require cancer-specific confirmation, and bacterial prognostic findings cannot be assumed to apply to fungi (Mima et al., 2016; Bullman et al., 2017; Yu T. et al., 2017; Nejman et al., 2020; Dohlman et al., 2022; Narunsky-Haziza et al., 2022). Apparent survival associations are readily confounded by stage, surgery, antibiotics, biliary or pancreatic stents, nutrition, cachexia, infection, and treatment intensity. A single postdiagnosis stool sample cannot distinguish a prognostic fungal state from a marker of advanced disease or clinical care.

In CRC, candidate fungal ratios and networks have not yet shown consistent, externally validated prognostic value after adjustment for tumor and treatment variables. No specific fungus has been shown to independently predict GC recurrence or survival. PDAC mechanisms suggest possible prognostic relevance, and tumor bacterial diversity has been linked to outcome, but neither observation establishes an independent fungal biomarker (Aykut et al., 2019; Riquelme et al., 2019; Zhong et al., 2021; Alam et al., 2022). Fungal models should be compared with stage, molecular subtype, treatment, immune state, nutrition, *H. pylori* status in GC, and established clinical prediction tools.

Longitudinal sampling before therapy, during treatment, after surgery, and at relapse is needed to separate tumor-associated change from antibiotics, proton pump inhibitors (PPIs), stents, nutritional support, chemotherapy, immunotherapy, hospitalization, or immune suppression. Within-patient trajectories may be more robust than between-person abundance differences, but their use for monitoring requires standardized timing, reproducible assays, and evidence that a change predicts recurrence or response early enough to alter management.

### 6.3 Treatment response and microbiome-based intervention

Bacterial microbiome studies link community composition and microbial metabolites to checkpoint-blockade response, but comparable fungal evidence in digestive system cancers is sparse and should not be inferred from melanoma or bacterial-only cohorts (Iida et al., 2013; Sivan et al., 2015; Vétizou et al., 2015; Zitvogel et al., 2015; Derosa et al., 2018; Gopalakrishnan et al., 2018; Matson et al., 2018; Routy et al., 2018; Pinato et al., 2019; Mager et al., 2020; Baruch et al., 2021; Davar et al., 2021; Spencer et al., 2021; McCulloch et al., 2022). Fungi could theoretically influence therapy through barrier injury, antigen presentation, myeloid remodeling, Th17 programs, complement, or cross-kingdom effects; however, these mechanisms are model- and context-dependent. Current evidence does not establish a fungal predictor of immunotherapy, chemotherapy, radiotherapy, or targeted-therapy response in CRC, GC, or PDAC.

Prospective treatment studies should collect pretreatment samples before antibiotics or bowel preparation when feasible and continue sampling at prespecified clinical time points. Fungal load, fungal and bacterial profiles, metabolites, immune phenotypes, diet, medication, and infection events should be analyzed together. Prediction models require locked analysis plans and external validation, and should test whether fungal information adds value beyond tumor biomarkers and bacterial-only models rather than merely correlating with response.

Chemotherapy, radiotherapy, mucosal injury, neutropenia, antibiotics, antifungals, nutritional change, and hospitalization can alter fungal ecology and infection risk (Viaud et al., 2013; Sokol et al., 2017; Suez et al., 2018; Zmora et al., 2018; Jiang et al., 2020; Seelbinder et al., 2023). These exposures are major confounders and plausible causes of observed dysbiosis, not background variables. Treatment-associated *Candida* expansion, for example, may reflect loss of bacterial colonization resistance or immune suppression rather than antitumor or protumor activity. Safety monitoring must distinguish ecological signals from invasive fungal disease in vulnerable patients.

Antifungal depletion and *Malassezia* recolonization changed tumor growth in mouse PDAC models (Aykut et al., 2019), providing causal experimental evidence but not a clinical treatment indication. Broad empiric antifungal therapy could disrupt commensal fungi, bacterial networks, colonization resistance, and drug metabolism, while selecting resistance or toxicity. fecal microbiota transplantation (FMT), probiotics, diet, precision antifungals, or host-pathway modulation therefore require a defined mechanism, a measurable target, a selected patient population, pharmacodynamic evidence, infection safeguards, and controlled trials (Hoffmann et al., 2013; David et al., 2014; Redman et al., 2014; Makki et al., 2018; Suez et al., 2018; Zmora et al., 2018; DeFilipp et al., 2019). No fungal-directed anticancer intervention is currently validated.

### 6.4 Designing mycobiome-aware translational studies

Translational studies must demonstrate analytical validity, clinical validity, and clinical utility in sequence. Fungal features should be compared with established tools: fecal testing and colonoscopic risk in CRC, *H. pylori* and premalignant histology in GC, and CA 19-9, imaging, cyst features, and pancreatitis status in PDAC (Zeller et al., 2014; Yu J. et al., 2017; Aykut et al., 2019; Riquelme et al., 2019; Thomas et al., 2019; Wirbel et al., 2019; Zhong et al., 2021; Kartal et al., 2022). Statistical significance or an internally cross-validated area under the curve is insufficient without calibration, external validation, incremental value, and a prespecified clinical action.

Sampling must match the biological question. Stool reflects luminal ecology but not necessarily mucosal, ductal, or tumor niches; biopsies and tumor tissue introduce preparation, carryover, processing, host-DNA, and low-biomass risks (Salter et al., 2014; Eisenhofer et al., 2019; Fletcher et al., 2023). Strong designs pair relevant compartments with extraction and environmental controls, absolute fungal-load measurements, bacterial profiling, and orthogonal localization. Multicenter protocols should standardize collection, storage, extraction, ITS target, sequencing depth, taxonomic database, batch handling, and contaminant removal before enrollment begins (Schoch et al., 2012; Salter et al., 2014; Knight et al., 2018; Eisenhofer et al., 2019; Nilsson et al., 2019a; Nilsson et al., 2019b).

Antibiotics, antifungals, PPIs, bowel preparation, chemotherapy, immunotherapy, stents, nutrition, diabetes, hospitalization, geography, and oral health can generate alternative explanations for fungal associations. Prospective protocols should predefine covariates and sampling windows, record indication and timing for each exposure, and avoid adjusting indiscriminately for variables that may lie on the causal pathway. Negative-control analysis, sensitivity analysis, and replication across centers are needed to assess confounding and reverse causation.

Precision models should integrate fungal features with bacterial taxa, metabolites, host immunity, tumor phenotype, diet, antimicrobials, and clinical variables (Iida et al., 2013; Zeller et al., 2014; Dzutsev et al., 2015; Brennan and Garrett, 2016; Gopalakrishnan et al., 2018; Matson et al., 2018; Routy et al., 2018; Nejman et al., 2020; Baruch et al., 2021; Davar et al., 2021; Sepich-Poore et al., 2021). *Candida*te fungal phenotypes must be defined by compartment, absolute load, activity, and evidence tier; a stool *Candida* signal after chemotherapy is not equivalent to spatially localized fungal activity in untreated tumor tissue. Parsimonious, interpretable models are preferable to high-dimensional signatures that cannot be reproduced or linked to a management decision.

Clinical development should be phased: reproducible observational phenotype; spatial and functional validation; assay locking and external validation; safety and biomarker-modulation studies; and only then efficacy testing in a selected population. This sequence limits unintended ecological effects and prevents premature antifungal intervention (Hoffmann et al., 2013; David et al., 2014; Redman et al., 2014; Makki et al., 2018; Suez et al., 2018; Zmora et al., 2018; DeFilipp et al., 2019). Trials should define whether the intervention targets a fungus, a fungal product, a host pathway, or a broader ecological state, because each strategy has different eligibility, monitoring, and safety requirements. The workflow is summarized in **Figure 4**.

**Figure 4:**
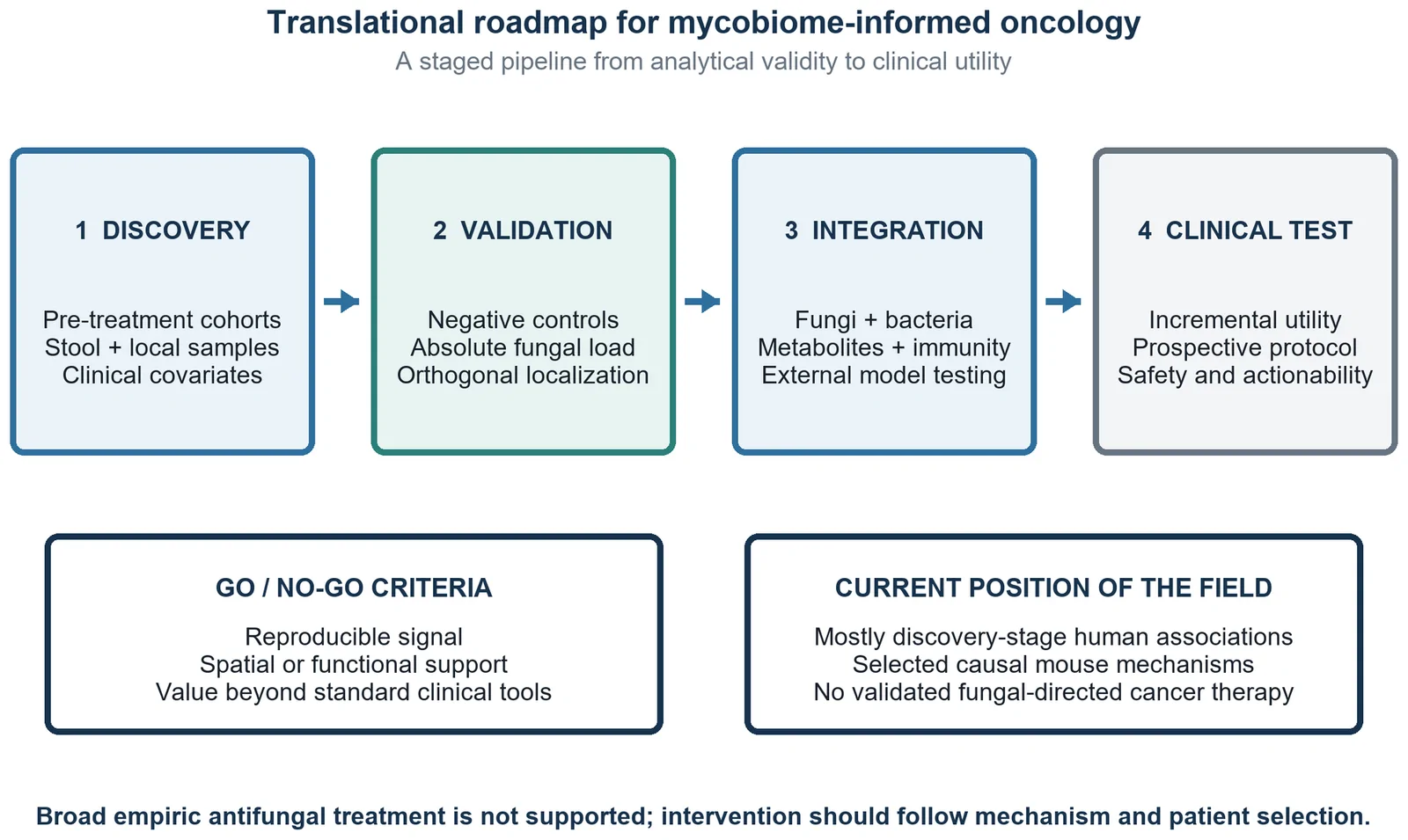
Staged translational roadmap for mycobiome-informed oncology. Translation should proceed from discovery to analytical and spatial validation, multi-omic integration, and prospective clinical testing. Advancement requires reproducibility, functional support, incremental value beyond standard clinical tools, and a clearly defined action. No fungal-directed cancer therapy is currently validated.

## 7 Discussion, limitations, and future perspectives

Low fungal biomass is the central analytical limitation, especially in pancreatic tissue, gastric mucosa, blood, and small biopsies. Reagent, environmental, skin, oral, and batch signals can dominate true biological signal (Salter et al., 2014; Eisenhofer et al., 2019; Fletcher et al., 2023). Relative-abundance shifts may also arise when bacterial biomass changes even if fungal load does not. Studies therefore require extraction blanks, no-template controls, mock communities, randomized processing, batch-aware analysis, absolute fungal-load measurements, independent replication, and *in situ*, culture-based, or other orthogonal confirmation where feasible.

Most human studies are cross-sectional and cannot distinguish cause from consequence. Reverse causation is plausible because tumors alter anatomy, oxygenation, pH, immunity, nutrition, medication exposure, obstruction, and barrier integrity. Alternative explanations include oral carryover, dietary fungi, PPIs, antibiotics, bowel preparation, stents, hospitalization, cachexia, and anticancer treatment. Small cohorts, multiple testing, center effects, and incomplete covariate measurement further inflate apparent specificity. Longitudinal premalignant-to-cancer cohorts, causal inference strategies, and paired functional models are therefore essential.

Cancer coverage is uneven: CRC, GC, and PDAC dominate, whereas esophageal, biliary, small-intestinal, hepatocellular, metastatic, and treatment-defined niches remain preliminary. Even within one cancer, stool, mucosa, tumor core, invasive front, ductal fluid, and metastases may represent different ecosystems. Findings should not be generalized across organs, populations, or specimen types without compartment-specific replication (Rao et al., 2022).

Spatial heterogeneity is underexamined. Stool cannot represent mucosal, ductal, stromal, necrotic, invasive-front, or metastatic niches, and bulk tissue cannot show whether fungi are extracellular, intracellular, viable, or adjacent to the responding host cells. Spatial microbiomics, fungal FISH, histology, laser-capture microdissection, culture where feasible, and matched host profiling should test localization and co-occurrence with epithelial injury or immune states. Spatial proximity strengthens plausibility but does not itself establish causality.

Host heterogeneity must be modeled explicitly. Age, sex, geography, diet, BMI, diabetes, oral health, nutrition, PPIs, antimicrobials, cancer therapy, and immune status can define both confounding and biologically relevant subgroups. Host genetics and fungal strain variation may also modify sensing and virulence. Integrative models must be interpretable, externally validated, and based on reproducible ecological states rather than isolated taxa; otherwise, apparent precision may represent cohort-specific overfitting.

### 7.1 Priorities for the next generation of studies

The first priority is reproducibility. Studies should report specimen type and timing, storage, extraction, ITS region and primers, sequencing depth, host-DNA handling, negative controls, fungal-load assay, database version, contaminant-removal strategy, and all major clinical exposures (Schoch et al., 2012; Salter et al., 2014; Knight et al., 2018; Eisenhofer et al., 2019; Nilsson et al., 2019a; Nilsson et al., 2019b). Multicenter ring trials and shared reference materials would determine whether the same specimen produces comparable results across laboratories. These details are not merely technical: they determine whether a low-biomass signal is credible and transferable.

The second priority is spatial and temporal validation. Fungal localization should be linked to host response, and repeated sampling should establish whether dysbiosis precedes, accompanies, or follows tumor progression and treatment. Matched stool, mucosa, tumor, adjacent tissue, and clinically relevant fluids can clarify compartment relationships. Spatial proximity strengthens plausibility but does not establish causality without functional evidence, while longitudinal association still requires control of time-varying treatment and disease severity (Fletcher et al., 2023).

The third priority is functional and strain-level resolution. Culture, isolate genomics, metatranscriptomics, metabolomics, organoids, defined co-cultures, gnotobiotic models, dose-response experiments, depletion-reconstitution, and host-pathway blockade can distinguish active organisms from detected DNA and test strain-specific effects (Gainza-Cirauqui et al., 2013; Mayer et al., 2013; Kashem et al., 2015; Moyes et al., 2016). Experiments should use clinically relevant abundance, anatomical conditions, and bacterial partners and should report whether a mechanism is necessary, sufficient, or merely permissive.

The fourth priority is actionability. A fungal signature must identify a population in whom management changes and add value beyond clinical, bacterial, metabolic, immune, imaging, and genomic variables. External and prospective validation are prerequisites. For interventions, investigators must define the target, eligibility biomarker, ecological and infection-related safety monitoring, resistance risk, and stopping rules. **Table 3** summarizes the major challenges and practical solutions.

**Table 3:** Major challenges and future directions in digestive system cancer mycobiome research.

Challenge	Why it matters	Recommended solution	Relevant setting
Low-biomass samples	Tissue, pancreatic, gastric, and blood samples may contain very low fungal burden and are vulnerable to background signal.	Include extraction blanks, reagent controls, fungal load quantification, and spatial localization.	PDAC; GC tissue studies; blood-based studies
Contamination	Environmental, reagent, and skin-associated fungi may produce false-positive signals.	Use batch randomization, contaminant identification, independent validation, and mock communities.	All tumor tissue mycobiome studies
Non-standardized ITS workflows	ITS1/ITS2 region, primer choice, sequencing depth, and bioinformatic pipelines affect fungal profiles.	Report primer set, ITS region, database version, filtering threshold, and quality-control pipeline.	Cross-cohort comparison
Incomplete fungal databases	Species-level annotation can be unstable, especially for low-abundance fungi.	Combine UNITE database annotation with shotgun metagenomics, culture, and quantitative PCR (qPCR) validation.	Species-level interpretation
Cross-sectional design	Most studies cannot distinguish cause, amplifier, biomarker, or consequence.	Develop longitudinal cohorts from premalignant lesions to cancer, treatment, recurrence, and metastasis.	CRC adenoma-carcinoma sequence; GC Correa cascade; PDAC progression
Insufficient causal evidence	Association does not prove fungal-driven tumor biology.	Use organoids, gnotobiotic animals, fungal depletion/recolonization, and pathway blockade.	Mechanistic studies
Limited spatial validation	Stool signals do not necessarily represent mucosal or tumor-localized fungi.	Apply FISH, IHC, spatial transcriptomics, and laser-capture microdissection.	GC and PDAC tissue studies
Host heterogeneity	Diet, age, sex, BMI, diabetes, antibiotics, PPIs, chemotherapy, immunotherapy, and geography affect fungal ecology.	Predefine covariates and perform stratified multivariable analysis.	Clinical cohorts
Uneven cancer-type coverage	CRC, GC, and PDAC dominate current evidence; esophageal, biliary, and small-intestinal tumors remain underexplored.	Treat less-studied cancers as future directions and conduct focused studies.	ESCC, biliary tract tumors, small-intestinal tumors
Clinical actionability	Fungal signatures are not yet standalone clinical tests.	Integrate fungal data with bacterial, metabolic, immune, imaging, and clinical variables; perform external validation.	Diagnosis, prognosis, treatment-response prediction

CRC, colorectal cancer; GC, gastric cancer; PDAC, pancreatic ductal adenocarcinoma; ESCC, esophageal squamous cell carcinoma; ITS, internal transcribed spacer; PPI, proton pump inhibitor; FISH, fluorescence *in situ* hybridization; IHC, immunohistochemistry; BMI, body mass index.

## 8 Conclusion

The gut mycobiome is a plausible but incompletely validated component of digestive system cancer ecology. Human CRC studies provide the broadest ecological evidence, whereas GC evidence remains predominantly cross-sectional and *Candida*-associated. In PDAC, human tissue studies support association, while the strongest causal evidence for *Malassezia*–mannose-binding lectin (MBL)–complement and fungal IL-33/type 2 pathways derives from mouse perturbation models. Fungal signals may be drivers, amplifiers, biomarkers, passengers, or consequences, and their meaning depends on anatomical compartment, fungal load, viability, host context, and treatment. Progress requires rigorous low-biomass controls, standardized and longitudinal sampling, spatial localization, cross-kingdom and functional validation, and external testing against established clinical tools. Until those requirements are met, fungal features should be treated as investigational additions to multi-omic oncology rather than standalone diagnostic tests or therapeutic targets.
